# Annual Scientific Retreat: Predisposing Factors in the Development of Complex Diseases

**Published:** 2005-04

The 2004 NIEHS Division of Extramural Research and Training’s annual scientific retreat was titled “Predisposing Factors in the Development of Complex Diseases.” The retreat focused on factors related to aging, immunology, infection, and genetics in the context of understanding risk modulators of environmentally induced diseases.

In the first session, participants discussed environmental influences on aging, including the contributions of environmental exposures to the development of chronic diseases, as well as new mouse models for aging and the validity of some of the more popular theories of aging in light of recent study results. The role of oxidative stress and accumulated oxidative damage as a factor in the development of chronic diseases was a common thread throughout these presentations.

The second session focused on innate immunity, inflammation, and the role of infection as predisposing factors in chronic disease. One presentation examined the role of bacterial load, asthma, and a hypothesized endotoxin switch that results in diametrically different effects in response to an antigen, depending on the dose and timing of exposure. Given that endotoxin potentiated the toxicity of several chemicals noted for their idiosyncratic reactions, these responses may have resulted from low-level, episodic, inflammatory events. Finally, data presented in the final talk of the session suggest that undernutrition in a host organism plays an important role in the rapid evolution of infectious agents including influenza viruses.

The final session focused on opportunities and challenges in using population-based studies to investigate gene–environment interactions leading to human disease, including the lack of repeatability of candidate gene association studies. A “unified” approach that assembles overlapping and synergistic networks of pathways for analyses was recommended in place of single candidate gene studies. This unified approach relies on data from expression microarrays, proteomics, and siRNA studies to identify genes that may not be part of recognized pathways. All relevant information can be analyzed using a hierarchical modeling approach.

One example given demonstrated some of the challenges of using animal models to study human diseases. Data from mouse models used to study atherogenesis displayed a paradox when considering genotype and phenotype. Contrary to the accepted hypothesis positing downregulation of low-density lipoprotein receptor (LDLR) in humans, expression of the human APOE4 isoform combined with increased LDLR was harmful in genetically modified mice when fed a high-fat Western diet, predicting important interactions between genotype/phenotype and exposure.

The topics of this year’s retreat offered potential new research directions by demonstrating some of the challenges in identifying predisposing factors in complex diseases. The Division of Extramural Research and Training will consider these issues as it develops strategic plans for enhancing research programs at the NIEHS.

## Invited Speakers

**Melinda Beck**

University of North Carolina–Chapel Hill

**Robert Floyd**

Oklahoma Medical Research Foundation

**John Groopman**

The Johns Hopkins University

**Howard Hu**

Harvard School of Public Health

**Nobuyo Maeda**

University of North Carolina–Chapel Hill

**Richard Miller**

University of Michigan School of Medicine

**Robert Roth**

Michigan State University

**Silke Schmidt**

Duke University Medical Center

**Duncan Thomas**

University of Southern California

**Holly Van Remmen**

The University of Texas Health Science Center at San Antonio

**Donata Vercelli**

The University of Arizona

